# Detection of gene variants associated with recessive limb–girdle muscular weakness and Pompe disease in a global cohort of patients through the application of next-generation sequencing analysis

**DOI:** 10.3389/fgene.2024.1477291

**Published:** 2024-11-29

**Authors:** Jorge Alfredo Bevilacqua, Abdullah Mohammed Al-Salti, Abubaker Al Madani, Armando Alves da Fonseca, Hacer Durmus, Josiah Chai, Ali Alshehri, Márcia Gonçalves Ribeiro, Paulo Sgobbi, Sergey S. Nikitin, Steven Vargas, Adriana Furtado, Nathan Thibault, Roberto Araujo, Nadia Daba

**Affiliations:** ^1^ Department of Neurology and Neurosurgery, Hospital Clínico Universidad de Chile, Santiago, Chile; ^2^ Department of Neurology, Neuroscience Center, Khoula Hospital, Muscat, Oman; ^3^ Department of Neurology, Rashid Hospital, Mohammed Bin Rashid University, Dubai, United Arab Emirates; ^4^ Department of Pathology and laboratory medicine, DLE Laboratory, Rio de Janeiro, Brazil; ^5^ Department of Neurology, Istanbul Faculty of Medicine, Istanbul University, Istanbul, Türkiye; ^6^ Department of Neurology, National Neuroscience Institute (TTSH Campus), Singapore, Singapore; ^7^ Neuromuscular Integrated Practice Unit, Neuroscience Center, King Faisal Specialist Hospital & Research Centre, Riyadh, Saudi Arabia; ^8^ Department of Pediatrics, Medical Consulting Sector of Personalized Medicine, DLE Laboratory, Rio de Janeiro, Brazil; ^9^ Department of Neurology and Neurosurgery, Federal University of São Paulo, São Paulo, Brazil; ^10^ Department of Genetics of Neurological Diseases, Research Centre for Medical Genetics, Moscow, Russia; ^11^ Department of Genetics of Neurological Diseases, National Institute of Neurology and Neurosurgery Manuel Velasco Suárez, Mexico City, Mexico; ^12^ Department of Global Rare Disease Scientific Affairs & Diagnostics, Sanofi, São Paulo, Brazil; ^13^ Sanofi Global Medical Affairs Rare Diseases, Sanofi, Cambridge, MA, United States; ^14^ Department of Evidence Generation Strategy for Rare Diseases, Sanofi, Cambridge, MA, United States

**Keywords:** next-generation sequencing, late-onset Pompe disease, limb-girdle muscular dystrophies, limb-girdle muscular weakness, acid α-glucosidase

## Abstract

**Introduction:**

Hereditary myopathies arise due to numerous pathogenic variants occurring in distinct genes, which amount to several hundred. Limb–girdle muscular dystrophies (LGMDs) constitute a heterogeneous group of neuromuscular disorders involving more than 30 genes. Clinically, LGMD is characterized by limb–girdle muscular weakness (LGMW). Late-onset Pompe disease is an important disorder with a differential diagnosis for LGMD, where next-generation sequencing (NGS) plays a crucial role in accurate and prompt diagnosis. The sensitivity and specificity of a 10-gene NGS panel have been previously evaluated for the prevalent forms of recessive LGMD (LGMD-R) and Pompe disease in Latin American patients with LGMW of unknown cause. This project aims to identify the regional relative prevalence of frequent LGMD-R subtypes and Pompe disease in a larger geographic area and to diagnose patients with LGMW by identifying genetic variants of LGMD-R and Pompe disease.

**Methods and Results:**

This 21-country multicentric analysis enrolled 2,372 patients with LGMW from 2017 to 2018. Sequencing analysis was performed using the Illumina NextSeq 500 system, and variant interpretation was performed according to the American College of Medical Genetics and Genomics guidelines. Pathogenic or likely pathogenic variants were seen in 11% of patients (n = 261). Among the positive cases, NGS effectively diagnosed 86.2% and 13.8% of patients with LGMD and Pompe disease, respectively. The most prevalent pathogenic acid α-glucosidase (*GAA)* variant identified was c.-32-13T > G.

**Conclusion:**

The study adds to the knowledge of the relative occurrence of various subtypes of LGMD worldwide. The inclusion of *GAA* in the NGS panel to investigate patients with LGMW is a powerful diagnostic approach to screen for late-onset Pompe disease.

## 1 Introduction

Hereditary myopathies encompass a broad spectrum of genetically determined muscular disorders caused by several hundred pathogenic variants in distinct genes (Benarroch et al., 2024). Among these, limb–girdle muscular dystrophies (LGMDs) comprise a diverse group of hereditary diseases distinguished by progressive limb–girdle muscular weakness (LGMW) and dystrophic features in the muscle biopsy (Nigro and Savarese, 2014; Khadilkar et al., 2018; Straub et al., 2018; Wicklund, 2019). Currently, there are 32 subtypes of LGMD. Depending on inheritance pattern, LGMD is classified into two main categories: autosomal dominant (LGMD-D) and autosomal recessive (LGMD-R). LGMD-D represents 10% of the LGMD, encompassing five subtypes (LGMD-D1 to D5), whereas 90% are LGMD-R, comprising 27 forms (LGMD-R1 to R27), each of which is caused by different pathogenic gene variants, with considerable phenotypical overlap (Straub et al., 2018; Wicklund, 2019; National Organization for Rare Disorders, 2021; Benarroch et al., 2024).

The diagnosis of a specific LGMD and obtaining appropriate prevalence estimates for each LGMD subtype are challenged by the low reported incidence rates of 1 in 14,500 to 1 in 123,000 (Liu et al., 2019; National Organization for Rare Disorders, 2021). Additionally, there is a huge phenotype overlap between LGMD and several other forms of LGMW (Nigro and Savarese, 2014; Khadilkar et al., 2018; Liu et al., 2019). Among these, Pompe disease is a rare, metabolic, autosomal recessive disease associated with the *GAA* (acid α-glucosidase) gene whose clinical signs and symptoms overlap with those of LGMD-R (Werneck et al., 2013; Lévesque et al., 2016; Lorenzoni et al., 2018). The estimated incidence of Pompe disease is 1 in 40,000 births in the United States (National Organization for Rare Disorders, 2021). Proximal muscle weakness of the shoulder and pelvic girdles (LGMW) and impairment of respiratory function are characteristic features of late-onset Pompe disease (LOPD) (Schüller et al., 2012; Lévesque et al., 2016).

The screening diagnostic method in patients suspected to have Pompe disease is an enzymatic analysis from dried blood spot (DBS). Molecular analysis confirms the biochemical diagnosis, and is especially useful for the early diagnosis of individuals with LGMW, including LGMD-R subtypes and Pompe disease (Reddy et al., 2017; Tsai et al., 2017; Bevilacqua et al., 2020). Next-generation sequencing (NGS), has induced a paradigm shift in the effective diagnosis of diseases with diverse phenotypes such as LGMD or LGMW (Málaga et al., 2019). As such, this study aimed to investigate the relative prevalence of LGMD-R and Pompe disease in a large cohort of individuals with genetically unclassified LGMW from 21 countries using a 10-gene NGS panel that includes nine genes for LGMD-R (*CAPN3*, *DYSF, SGCA*, *SGCB*, *SGCD*, SGCG, *FKRP*, *ANO5*, *TCAP)* and the gene for Pompe disease (*GAA).* The secondary objective of this study was to identify the region-wise relative prevalence of LGMD-R forms and Pompe disease to create awareness about the overlapping phenotype of Pompe disease with LGMD.

## 2 Materials and methods

### 2.1 Sample

This multicenter investigation included a large collection of samples from 21 countries, encompassing Brazil, Mexico, Colombia, Russia, Malaysia, Turkey, Saudi Arabia, Israel, Oman (Gulf), Panama, Peru, Chile, Hong Kong, Kazakhstan, South Africa, Singapore, Dominican Republic, Costa Rica, Ecuador, Guatemala, and El Salvador. Global epidemiology, countrywide and region-wide prevalence, and local technical logistic capacity were considered for the selection of the countries. The project was designed and conducted as per the Declaration of Helsinki and the International Conference on Harmonization Good Clinical Practice Guidelines. IPPMG at the Universidade Federal do Rio de Janeiro approved the research with number 3.642.024.

The inclusion criteria for patients were as follows: male and female patients aged 5 years and above; with progressive LGMW without definitive diagnosis per molecular and/or immunohistochemical analysis; with or without elevated serum creatine kinase (CK) activity; and not previously tested for Pompe disease (enzyme activity or *GAA* genetic testing). Patients with Pompe disease with a previous positive test (enzyme activity or *GAA* genetic testing) were excluded from the study.

### 2.2 Gene panel testing procedures

The gene panel was prescribed by a neuromuscular specialist in accordance with the clinical inclusion criteria, but specific clinical data or phenotype descriptions of the patients were not collected as part of this study. Genomic DNA was extracted from peripheral dried blood spots of each patient and collected on filter paper for molecular testing. The samples (obtained by convenience sampling) were received between 2017 and 2018. The 10-gene NGS panel was developed at the Diagnósticos Laboratoriais Especializados (DLE), Sao Paulo, Brazil. Further NGS testing was processed at the DLE as described previously (Bevilacqua et al., 2020). The NGS panel was chosen based on worldwide prevalence, national and regional epidemiology, and local technical capacity. It comprised nine recessive genes for LGMD (*CAPN3*, *DYSF, SGCA*, *SGCB*, *SGCD*, SGCG, *FKRP*, *ANO5*, *TCAP)* and one gene for Pompe disease (*GAA*) (Supplementary Table S1) (Straub et al., 2018; Wicklund, 2019; Benarroch et al., 2020; Bevilacqua et al., 2020; Schiava et al., 2022).

### 2.3 Sequencing analysis and variant interpretation

Variants were classified based on the American College of Medical Genetics and Genomics as “pathogenic,” “likely pathogenic,” “variant of uncertain significance,” “likely benign,” or “benign” with respect to a disease and its inheritance pattern (Ellard et al., 2020). Targeted NGS was performed with more than 98% coverage of target regions at 20X or greater for each gene in the panel using Agilent SureSelect^QXT^ target enrichment. Specific deep intronic variants were targeted, and sequencing of flanking intron/exon regions up to 25 base pairs was performed. A custom SureSelect^QXT^ kit (Agilent Technology) was used for the enrichment of these coded and flanked intronic regions. The Illumina NextSeq 500 system was used for sequencing these enriched regions as previously described (Bevilacqua et al., 2020).

### 2.4 NGS data and quality analysis

Following DNA sequencing, the sequence alignment was conducted using the Burrows–Wheeler Aligner software. This process involved analyzing the sequencing data by comparing it to the standard genome GRCh37 (hg19). The aligned data were used for variant calling (single-nucleotide variants and indels) with the SAMtools software, and annotation was performed using Ensembl Variant Effect Predictor (Bevilacqua et al., 2020).

To ensure accurate sample identification and obtain high-quality DNA, quality control (QC) procedures were conducted alongside the sequencing analysis. Essential steps of NGS, such as library preparation, target capturing, and data sequencing, underwent thorough quality checks. Additionally, mapping, and variant QC metrics were calculated for the sequencing output, allowing the exclusion and reevaluation of any failed samples. QualiMap software was used to check the quality of the sequencing analysis and call of variants. The data are expressed in percentages (Bevilacqua et al., 2020).

## 3 Results

### 3.1 Demographics

This study enrolled 2,372 patients across 21 countries between 2017 and 2018. The countries that participated in the study are shown in Supplementary Figure S1. Most of the patients were adults (≥18 years [57%]). The country-wise distribution of the enrolled patients and the number of patients who were positive for the studied LGMD subtypes and Pompe disease are shown in Supplementary Table S2.

### 3.2 Molecular diagnosis of LGMD-R and Pompe disease

**FIGURE 1:**
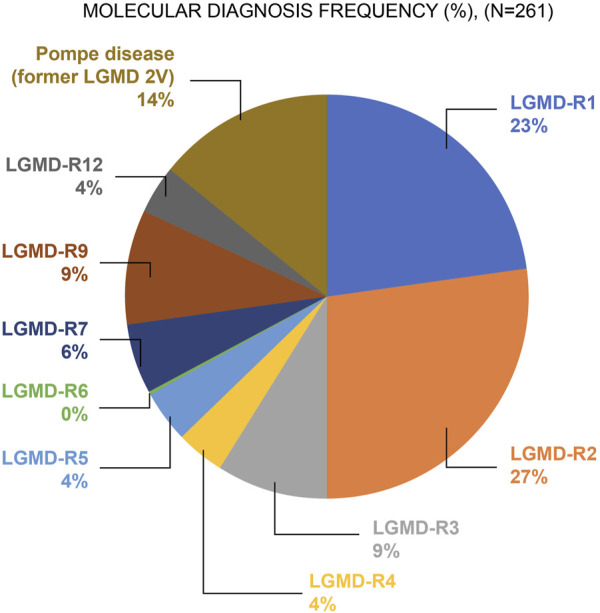
Frequency of individuals with confirmed LGMD-R subtype and Pompe disease (N = 261) Abbreviations: LGMD: Limb–girdle muscular dystrophy; LGMD-R: Recessive limb–girdle muscular dystrophy.

Of the 2,372 patients, 11% (n = 261) tested positive for LGMD-R or Pompe disease. Among the 261 positive cases, 86.2% (n = 225) of the patients had LGMD-R, and 13.8% (n = 36) were diagnosed with Pompe disease. Figure 1 shows the frequency of patients with LGMD-R subtypes and Pompe disease among the 261 confirmed cases. Of the enrolled population, the majority had LGMD-R2 (dysferlinopathy; 27%, n = 69), whereas LGMD-R6 (δ-sarcoglycanopathy; <1%, n = 1) (from Brazil) was the least commonly reported subtype.

The regional relative prevalence of LGMD-R subtypes across the 21 countries that participated in the study is represented in Supplementary Figure S2. Of the 69 patients diagnosed with LGMD-R2, the highest number of cases (n = 26) was reported from Brazil, followed by Mexico (n = 11), Turkey (n = 9), and Saudi Arabia (n = 8).

The frequencies of the most prevalent LGMD-R subtypes in 14 participating countries are presented in Supplementary Figure S3. LGMD-R2 was observed in the highest number of patients (n = 26 of 83 patients; the total screened population was 697) in Brazil.

### 3.3 Pompe disease in different age groups of the enrolled population

Pompe disease was the third most frequent cause of LGMW in the cohort, reported in 36 (13.8%) patients. Table 1 displays the age groups of the patients with Pompe disease. Although only male and female patients aged ≥5 years were considered based on the study inclusion criteria, these younger patients (n = 3) were included and screened as an exception because of special requests from healthcare professionals. Pompe disease was reported in 11 countries, with the highest number of cases from Brazil (n = 11). However, no patients with Pompe disease were reported from the remaining 10 countries (Colombia, Malaysia, Oman [Gulf], Panama, Chile, Hong Kong, Kazakhstan, Dominican Republic, Costa Rica, and Ecuador).

**TABLE 1:** Percentage of patients with Pompe disease in different age groups.

Age group	Pompe-positive patients, n (%)
<6 years^a^	3 (8.3%)
6–15 years	9 (25%)
16–25 years	5 (13.9%)
>26 years	19 (52.8%)

^a^ The age of the youngest patients with Pompe disease was below 6 years. Although only male and female patients aged 5 years and above were considered in the study, younger patients were included and screened as an exception because of special requests from healthcare professionals.

### 3.4 Frequency of *GAA* variants in the enrolled population

The commonly encountered pathogenic *GAA* variant identified in the population was c.-32-13T > G, which was detected in a heterozygous state at a frequency of 20%. The other *GAA* variants detected included c.2238G > C and c.2417C > T at a frequency of 5% each. The c.119G > A variant was identified in 4% of the cases, whereas c.1343G > C and c.1552-3C > G were identified in 3% of the cases.

## 4 Discussion

LGMD is a diverse group of neuromuscular diseases with substantial phenotypical overlap with other forms of myopathy, including Pompe disease, manifesting as LGMW (National Organization for Rare Disorders, 2021). Although more than 30 LGMD-associated genes are known, many affected patients remain undiagnosed because of the current challenges associated with gene detection methods and clinical overlap with other forms of myopathy-producing LGMW (Wicklund, 2019; National Organization for Rare Disorders, 2021). This diagnostic NGS project provides insights into the relative prevalence of the most common LGMD-R subtypes and Pompe disease across 21 countries, in some of which, there is no access to genetic diagnosis. In this study covering 2,372 patients, 225 patients were diagnosed with LGMD and 36 with Pompe disease through NGS. The findings of this study underscore the significance of genetic testing in the diagnosis of various disorders that present similar clinical symptoms.

The benefit of the gene panel in identifying genetic variants was reported with the inclusion of the *GAA* gene because 5% of the total population (121/2,372 individuals) were found to harbor *GAA* variants. Molecular diagnosis would have been missed without the inclusion of *GAA* in the gene panel. The most common *GAA* variant identified in this study was c.-32-13T>G (Supplementary Tables S3, S4).

Thirty-six (13.8%) patients from 11 countries, including Brazil, Mexico, Russia, Turkey, Saudi Arabia, Israel, Peru, South Africa, Singapore, Guatemala, and El Salvador, were diagnosed with Pompe disease. Interestingly, even among the countries that enrolled a small number of patients, such as Peru, South Africa, Singapore, Guatemala, and El Salvador, patients with Pompe disease were identified among those with LGMW.

The LOPD prevalence in a large group of patients in the United States, analyzed using a 35-gene NGS panel, was 0.8% (38/4,656 cases) (Nallamilli et al., 2018), whereas the LOPD prevalence in our previous Latin American study was 0.4% (9/2,103 cases) (Bevilacqua et al., 2020). The relative prevalence of 1.5% (36/2,372 individuals) was higher than the reported prevalence percentages. Therefore, promoting genetic testing for LGMD and Pompe disease among a representative global population is urgently needed for early disease management and awareness creation regarding the overlapping phenotypes of Pompe with LGMD and other forms of LGMW.

Pathogenic variants of the *CAPN3* gene cause calpainopathy (LGMD-R1), which is typically observed in patients aged 2–40 years (Nallamilli et al., 2018). In this study, a greater number of pathogenic variants were observed in the *DYSF* and *CAPN3* genes. This result indicated that allelic heterogeneity was higher in these genes than in the other genes in the panel. This study shows that dysferlinopathy and calpainopathy are frequently occurring LGMD-R subtypes in the enrolled population (*DYSF*-associated LGMD-R2: 69/261 [26.44%] and *CAPN3*-associated LGMD-R1:61/261 [23.37%]). These findings are consistent with those of Nallamilli et al. (2018) *,* who conducted a study on 4,656 patients with clinically suspected LGMD across the United States (LGMD-R1: 214/1259 LGMD-positive cases [17%]; LGMD-R2: 201/1259 LGMD-positive cases [16%]; N = 4,656) (Nallamilli et al., 2018) and the observations in our previous Latin American study (LGMD-R1: 90/335 LGMD-positive cases [26.87%]; LGMD-R2: 127/335 LGMD-positive cases [37.91%]; N = 2,103) (Bevilacqua et al., 2020). It is worth mentioning that the actual prevalence of LGMD-R2 may be much higher in some countries included and may not be represented in the present study as some patients with LGMD-R2 were included in the Latin American pilot study (37.91%) (Bevilacqua et al., 2020; Cerino et al., 2022) and ongoing studies.

To our knowledge, this is the first attempt at region-wise LGMD mapping across the 21 participating countries some of which did not have access to NGS testing. The results of this study are consistent with those of NGS projects conducted across different geographic regions. Nallamilli et al. (2018) and Töpf et al. (2020) conducted NGS testing among 4,656 North American patients and 1,001 European and Middle Eastern patients using a 35-gene panel and a 170-gene panel, respectively (Nallamilli et al., 2018; Töpf et al., 2020). Both studies included patients with undiagnosed LGMW and/or hyperCKemia. Moreover, 8 of the 12 genes in the study by Nallamilli et al. (2018)—*ANO5, SGCA, CAPN3, FKRP, SGCB, SGCB, DYSF,* and *GAA* (Nallamilli et al., 2018)—were part of the 10-gene panel used in the present study.

### 4.1 Limitations

The first limitation of this study is the low diagnostic yield, which may be due to a lack of proper understanding of the gene panel and the inclusion of a limited number of genes in the panel. Future research using gene panels with a greater number of genes can contribute to expanding the knowledge in this area. The limited patient population, particularly in certain countries, could be considered as the second limitation of this study. Additionally, the ethnicity of the patients could not be considered because of the unavailability of relevant information. The third limitation of this study was that disease severity in relation to genetic variants was not included. Further research on the validation and optimization of NGS panels, consideration of confounding factors, and a more comprehensive representation of diverse populations, including ethnicity and specific clinical findings, is necessary. Although NGS testing offers improved diagnostic capabilities, the long turnaround time must be considered. The period from the date that a laboratory received the testing kit from different countries to the final NGS result was 20 days.

## 5 Conclusion

The findings from this study reveal the benefits of the NGS approach in the genetic testing of LGMD and Pompe in a patient population of 2,372 from 21 countries. A confirmed diagnosis of LGMD-R or Pompe disease was made in 11% of the patients through NGS. Pompe disease was identified in 13.8% of the total diagnosed population, including patients from 11 participating countries. The most commonly identified *GAA* variant was c.-32-13T>G. NGS is a reliable test for the identification of patients with muscular weakness given that access to NGS technology and expertise is not a challenge. Hence, we propose that NGS be employed as a frontline diagnostic tool in individuals with suspected muscular diseases of genetic origin.

## Data Availability

The datasets presented in this study can be found in online repositories. The names of the repository/repositories and accession number(s) can be found below: https://d19rvjna3xg7q0.cloudfront.net/data/consolidado.tsv, FL00001.

## Abbreviations

CK, Creatine kinase; D, Dominant; DLE, Diagnósticos Laboratoriais Especializados; GAA, Acid α-glucosidase; LGMD, Limb–girdle muscular dystrophy; LGMW, Limb–girdle muscular weakness; LOPD, Late-onset Pompe disease; NGS, Next-generation sequence; QC, Quality control; R, Recessive.
