# Supplementary figures and images for: Detection of gene variants associated with recessive limb–girdle muscular weakness and Pompe disease in a global cohort of patients through the application of next-generation sequencing analysis
Source: Front Genet. 2024 Nov 29;15:1477291. doi: 10.3389/fgene.2024.1477291 (PMC11638199; doi:10.3389/fgene.2024.1477291)

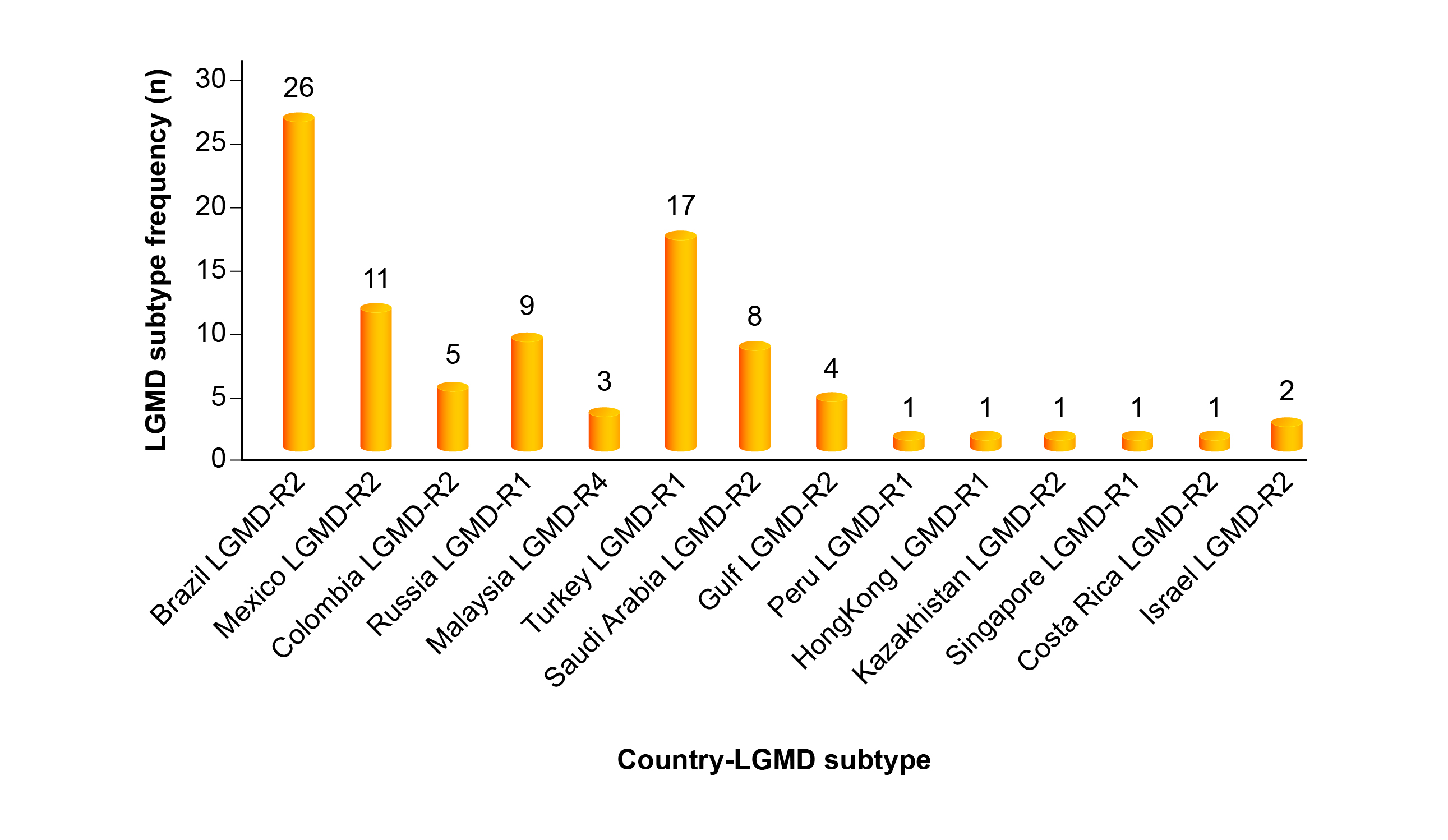

Supplement: Supplementary file 1 [file Image3.jpeg]

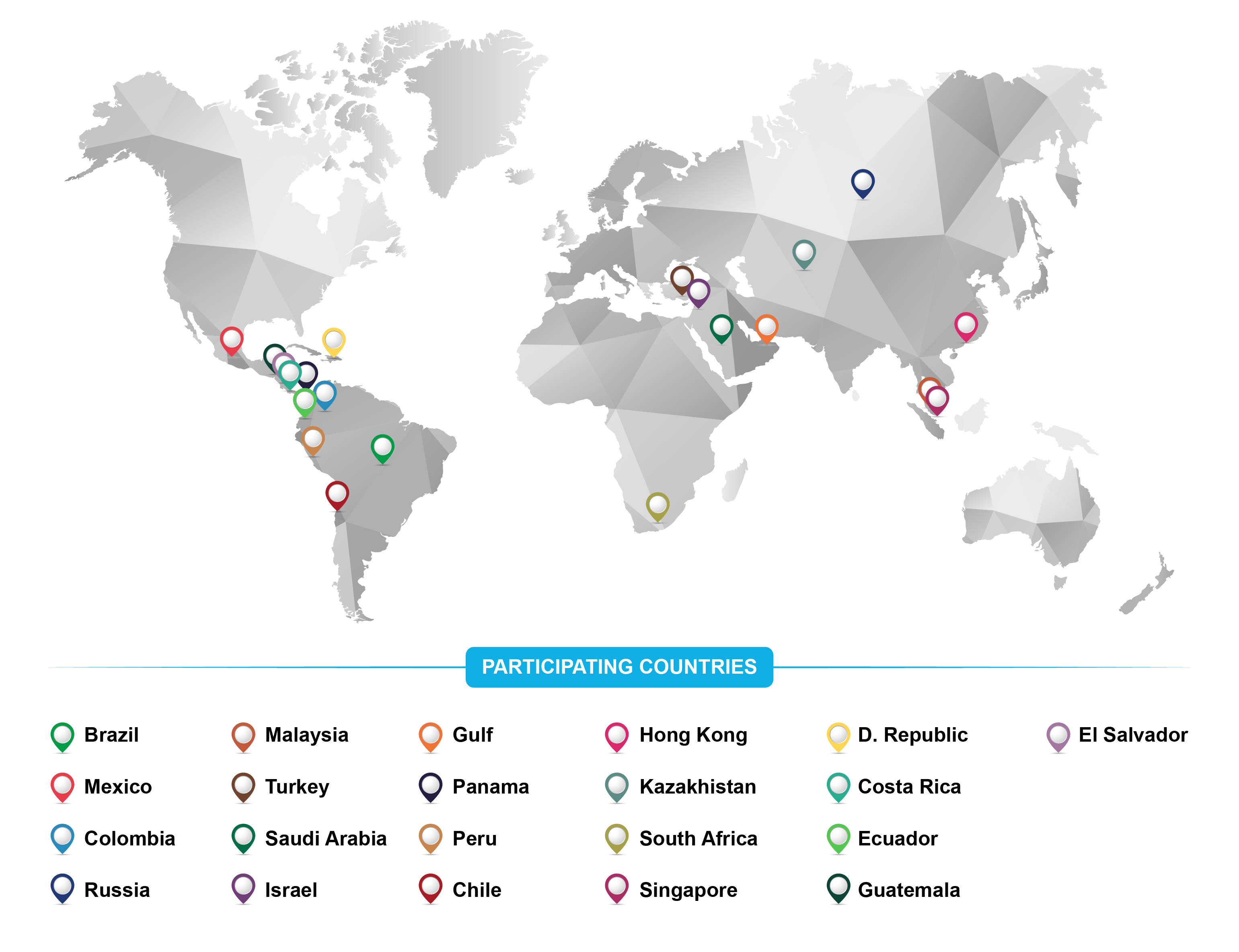

Supplement: Supplementary file 3 [file Image1.jpeg]

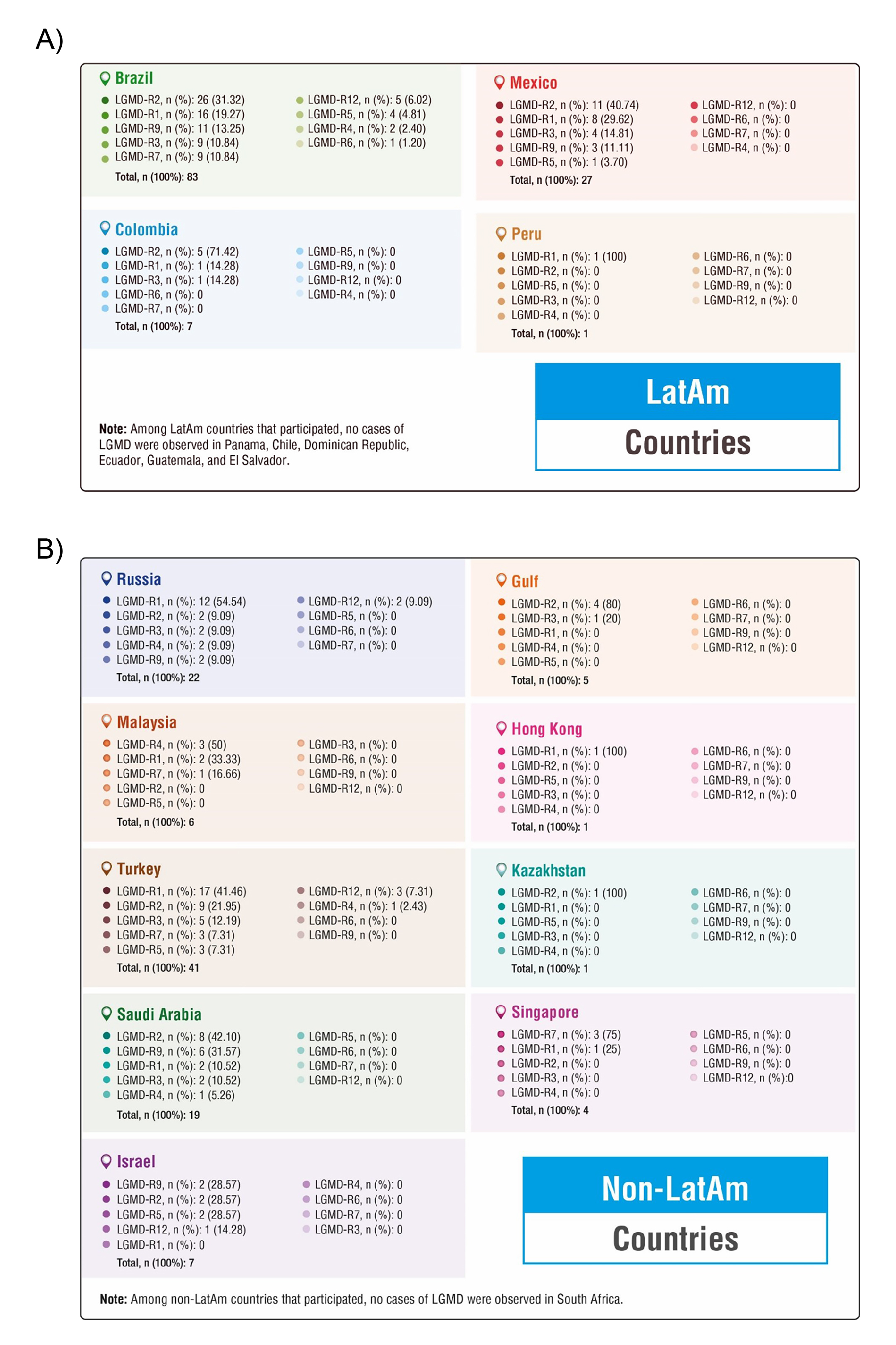

Supplement: Supplementary file 4 [file Image2.jpeg]
