# Supplementary material for: Detection of gene variants associated with recessive limb–girdle muscular weakness and Pompe disease in a global cohort of patients through the application of next-generation sequencing analysis
Source: Front Genet. 2024 Nov 29;15:1477291. doi: 10.3389/fgene.2024.1477291 (PMC11638199; doi:10.3389/fgene.2024.1477291)
Supplement: Supplementary file 2 [file Table1.docx]

**Supplementary Information**

**Supplementary Table 1.** Myopathies included in the gene panel (Benarroch et al., 2020; Straub et al., 2018; Wicklund, 2019)

| **Old nomenclature** | **New nomenclature** | **Affected protein name** | **Gene** |
| --- | --- | --- | --- |
| LGMD 2A | LGMD-R1 | Calpain 3 | *CAPN3* |
| LGMD 2B | LGMD-R2 | Dysferlin | *DYSF* |
| LGMD 2D | LGMD-R3 | α-Sarcoglycan | *SGCA* |
| LGMD 2E | LGMD-R4 | β-Sarcoglycan | *SGCB* |
| LGMD 2C | LGMD-R5 | γ-Sarcoglycan | *SGCG* |
| LGMD 2F | LGMD-R6 | δ-Sarcoglycan | *SGCD* |
| LGMD 2G | LGMD-R7 | Telethonin | *TCAP* |
| LGMD 2I | LGMD-R9 | Fukutin-related protein | *FKRP* |
| LGMD 2L | LGMD-R12 | Anoctamin 5 | *ANO5* |
| LGMD 2V | Pompe disease | Acid α-glucosidase | *GAA* |

*LGMD: Limb–girdle muscular dystrophy; LGMD-R: Recessive limb–girdle muscular dystrophy*

**Supplementary Table 2.** Summary statistics of the country-wise enrollment of patients with positive LGMD subtypes

| **Country** | **Total no. of patients screened/country, n, (% of positive patients)** | **LGMD-R–positive patients, n (%)** | **Pompe-positive patients, n (%)** |
| --- | --- | --- | --- |
| Brazil | 697, (13.48) | 83 (11.90) | 11 (1.57) |
| Mexico | 384, (7.55) | 27 (7.03) | 2 (0.52) |
| Colombia | 151, (4.63) | 7 (4.63) | 0 |
| Russia | 439, (7.06) | 22 (5.01) | 9 (2.05) |
| Malaysia | 32, (18.75) | 6 (18.75) | 0 |
| Turkey | 198, (22.72) | 41 (20.70) | 4 (2.02) |
| Saudi Arabia | 158, (13.91) | 19 (12.02) | 3 (1.89) |
| Israel | 94, (8.5) | 7 (7.44) | 1 (1.06) |
| Gulf | 39, (12.82) | 5 (12.82) | 0 |
| Panama | 19, (0) | 0 | 0 |
| Peru | 20, (10) | 1 (5) | 1 (5) |
| Chile | 8, (0) | 0 | 0 |
| Hong Kong | 22, (4.54) | 1 (4.54) | 0 |
| Kazakhstan | 18, (5.55) | 1 (5.55) | 0 |
| South Africa | 10, (10) | 0 | 1 (10) |
| Singapore | 24, (24.99) | 4 (16.66) | 2 (8.33) |
| Dominican Republic | 22, (0) | 0 | 0 |
| Costa Rica | 15, (6.66) | 1 (6.66) | 0 |
| Ecuador | 4, (0) | 0 | 0 |
| Guatemala | 11, (9.09) | 0 | 1 (9.09) |
| El Salvador | 7, (14.28) | 0 | 1 (14.28) |
| Total | 2372, (11.00) | 225 (9.49) | 36 (1.52) |

*LGMD: Limb–girdle muscular dystrophy; LGMD-R: Recessive limb–girdle muscular dystrophy.*

**Supplementary Table 3.** *GAA* variants observed in the enrolled population.

| ***GAA* variants** | **Frequency (n)** |
| --- | --- |
| **dnagaa1** | |
| -32-13T>G | 20 |
| 2238G>C | 5 |
| 2417C>T | 5 |
| 119G>A | 4 |
| 1343G>C | 3 |
| 1552-3C>G | 3 |
| 1352C>G | 2 |
| 1909C>A | 2 |
| 1935C>A | 2 |
| 266G>A | 2 |
| 2732C>T | 2 |
| -32-13T | 2 |
| 32G>A | 2 |
| 420C>A | 2 |
| 664G>A | 2 |
| 693-4G>T | 2 |
| 922C>T | 2 |
| 1000G>A | 1 |
| 1048G>A | 1 |
| 1198G>A | 1 |
| 1316T>A | 1 |
| 134C>G | 1 |
| 1388G>A | 1 |
| 1409A>C | 1 |
| 1417G>A | 1 |
| 1464dupC | 1 |
| 1607A>G | 1 |
| 1692T>G | 1 |
| 1757C>T | 1 |
| 1828G>A | 1 |
| 43G>A | 1 |
| 533G>A | 1 |
| 2741_2765dupAGCAGGTCCTCTCCAACGGTGTCCC | 1 |
| 1888G>A | 1 |
| 1905C>A | 1 |
| 1924G>A | 1 |
| 1927G>A | 1 |
| 1979G>A | 1 |
| 2011A>G | 1 |
| 2033T>C | 1 |
| 2051C>T | 1 |
| 2110G>A | 1 |
| 2114T>C | 1 |
| 212A>G | 1 |
| 2132C>G | 1 |
| 2239G>T | 1 |
| 2245G>T | 1 |
| 2275G>A | 1 |
| 2348T>G | 1 |
| 2432T>G | 1 |
| 257C>G | 1 |
| 2609G>A | 1 |
| 576G>C | 1 |
| 277G>A | 1 |
| 288G>C | 1 |
| 317G>A | 1 |
| -32-13T> | 1 |
| -32-18C>G | 1 |
| -32-3C>A | 1 |
| -32-3C>T | 1 |
| -32-9C>G | 1 |
| 352C>G | 1 |
| 370C>G | 1 |
| 502C>T | 1 |
| 569G>A | 1 |
| 631G>A | 1 |
| 685C>T | 1 |
| 688G>A | 1 |
| 688G>T | 1 |
| 752C>G | 1 |
| 826A>T | 1 |
| 841C>T | 1 |
| 862G>A | 1 |
| 910G>A | 1 |
| 956A>G | 1 |
| NA | 1 |
| **dnagaa2** | |
| 1927G>A | 2 |
| 2104C>T | 2 |
| 2275G>A | 2 |
| 1352C>G | 1 |
| 1655T>C | 1 |
| 1735G>A | 1 |
| 1905C>A | 1 |
| 1905C>G | 1 |
| 1941C>G | 1 |
| 2323C>A | 1 |
| 2560C>T | 1 |
| 258delC | 1 |
| 2725G>A | 1 |
| 307T>G | 1 |
| 693-4G>T | 1 |
| **dnagaa3** | |
| 2323C>A | 2 |
| 2408A>G | 1 |

*GAA*: Acid α-glucosidase.

**Supplementary Table 4.** Description of *GAA* variants identified in patients diagnosed with Pompe disease.

| Countries | *GAA* state | *GAA* class | DNA variant: Nucleotide change | N |
| --- | --- | --- | --- | --- |
| Brazil | Heterozygous | Pathogenic | -32-13T>G | 8 |
| Brazil | Heterozygous | Pathogenic | -32-3C>A | 1 |
| Brazil | Heterozygous | Pathogenic | -32-13T>G | 1 |
| Brazil | Heterozygous | VUS | 1352C>G | 1 |
| Mexico | Heterozygous | Pathogenic | -32-13T>G | 1 |
| Mexico | Heterozygous | Pathogenic | 1048G>A | 1 |
| Russia | Heterozygous | Pathogenic | -32-13T>G | 1 |
| Russia | Heterozygous | VUS | 2417C>T | 1 |
| Russia | Heterozygous | Pathogenic | 1316T>A | 1 |
| Russia | Heterozygous | Pathogenic | -32-13T>G | 2 |
| Russia | Heterozygous | Pathogenic | 1000G>A | 1 |
| Russia | Heterozygous | VUS | 119G>A | 2 |
| Russia | Heterozygous | VUS | 2011A>G | 1 |
| Saudi Arabia | Homozygous | Pathogenic | 1979G>A | 1 |
| Saudi Arabia | Heterozygous | VUS | 2275G>A | 1 |
| Saudi Arabia | Heterozygous | Pathogenic | -32-13T>G | 1 |
| Turkey | Heterozygous | VUS | 2732C>T | 1 |
| Turkey | Heterozygous | VUS | 266G>A | 1 |
| Turkey | Heterozygous | Pathogenic | 1464dupC | 1 |
| Turkey | Heterozygous | VUS | 420C>A | 1 |
| Israel | Heterozygous | VUS | 664G>A | 1 |
| Peru | Heterozygous | VUS | 2348T>G | 1 |
| South Africa | Heterozygous | VUS | 2110G>A | 1 |
| Singapore | Heterozygous | VUS | -32-9C>G | 1 |
| Singapore | Heterozygous | VUS | 862G>A | 1 |
| Guatemala | Heterozygous | Pathogenic | 2741_2765dupAGCAGGTCCTCTCCAACGGTGTCCC | 1 |
| El Salvador | Heterozygous | Pathogenic | -32-13T>G | 1 |

*DNA: Deoxyribonucleic acid; GLA: 𝛼-galactosidase A gene; VUS: Variant of unknown significance.*

**Supplementary Figure Legends**

**Supplementary Figure 1.** List of countries that participated in the project.

**Supplementary Figure 2.** Distribution of LGMD-R subtypes among the enrolled population- A) in Latin American countries; B) in non-Latin American countries.

*LGMD-R****:*** *Recessive limb–girdle muscular dystrophy.*

**Supplementary Figure 3.** Frequencies of the most prevalent LGMD-R subtype in different countries.

*LGMD: Limb–girdle muscular dystrophy; LGMD-R: Recessive limb–girdle muscular dystrophy.*
