# Supplementary material for: Detection of gene variants associated with recessive limb–girdle muscular weakness and Pompe disease in a global cohort of patients through the application of next-generation sequencing analysis
Source: Front Genet. 2024 Nov 29;15:1477291. doi: 10.3389/fgene.2024.1477291 (PMC11638199; doi:10.3389/fgene.2024.1477291)
Supplement: Supplementary file 5 [file Table2.docx]

Name- Muscular dystrophy

Number- FL00001

Link: <https://d19rvjna3xg7q0.cloudfront.net/data/consolidado.tsv>
